# Rural communities experience higher radon exposure versus urban areas, potentially due to drilled groundwater well annuli acting as unintended radon gas migration conduits

**DOI:** 10.1038/s41598-024-53458-6

**Published:** 2024-02-26

**Authors:** Selim M. Khan, Dustin D. Pearson, Evangeline L. Eldridge, Tiago A. Morais, Marvit I. C. Ahanonu, M. Cathryn Ryan, Joshua M. Taron, Aaron A. Goodarzi

**Affiliations:** 1 Department of Biochemistry & Molecular Biology, Robson DNA Science Centre, Arnie Charbonneau Cancer Institute, Cumming School of Medicine, University of Calgary, Calgary, AB Canada; 2 Department of Oncology, Arnie Charbonneau Cancer Institute, Cumming School of Medicine, University of Calgary, Calgary, AB Canada; 3 Department of Earth, Energy and Environment, Faculty of Science, University of Calgary, Calgary, AB Canada; 4 School of Architecture, Planning, and Landscape, University of Calgary, Calgary, AB Canada

**Keywords:** Environmental impact, Risk factors, Hydrogeology

## Abstract

Repetitive, long-term inhalation of radioactive radon gas is one of the leading causes of lung cancer, with exposure differences being a function of geographic location, built environment, personal demographics, activity patterns, and decision-making. Here, we examine radon exposure disparities across the urban-to-rural landscape, based on 42,051 Canadian residential properties in 2034 distinct communities. People living in rural, lower population density communities experience as much as 31.2% greater average residential radon levels relative to urban equivalents, equating to an additional 26.7 Bq/m^3^ excess in geometric mean indoor air radon, and an additional 1 mSv/year in excess alpha radiation exposure dose rate to the lungs for occupants. Pairwise and multivariate analyses indicate that community-based radon exposure disparities are, in part, explained by increased prevalence of larger floorplan bungalows in rural areas, but that a majority of the effect is attributed to proximity to, but not water use from, drilled groundwater wells. We propose that unintended radon gas migration in the annulus of drilled groundwater wells provides radon migration pathways from the deeper subsurface into near-surface materials. Our findings highlight a previously under-appreciated determinant of radon-induced lung cancer risk, and support a need for targeted radon testing and reduction in rural communities.

## Introduction

Radioactive radon (^222^Rn) exposure is an indoor air environmental carcinogen that is among the most prevalent non-tobacco causes of lung cancer, alongside 2.5-micron particulate matter (PM_2.5_) air pollutants, arsenic metalloids, and severe lung inflammation from infectious disease and/or silicate exposure^1–11^. The inhalation of gaseous radon and its solid radioactive progeny increases the relative lifetime risk of lung cancer by emitting alpha particle radiation that damages lung epithelial cell DNA, raising the likelihood of cancer-causing genetic mutations^12–16^. Radioactivity from decaying radon (and radon progeny) in indoor air is measured in Becquerels (Bq), equating with one alpha particle emission per second, per cubic metre (m^3^) of air, with a total of four alpha particle emissions occurring as ^222^Rn decays into stable ^210^Pb over time^16^. For large populations, there is a 16% increase in relative lifetime risk of lung cancer per 100 Bq/m^3^ of radon exposure that, based on the documented activity pattern of a typical North American adult, equates to an absorbed dose of ~ 4 millisieverts (mSv) per year (mSv/y) of particle radiation^17–21^. Understanding excess radon exposure is important because a substantial number of people diagnosed with lung cancer that do not meet current inclusion criteria for early cancer detection programs (e.g., 40% of Canadian patients), as they have used tobacco to only a limited extent (~ 20% of patients), or have never used tobacco at all (another ~ 20% of patients)^22–28^.

At a population and individual level, radon exposure arises from a complex convergence of geologic, built environment, demographic, lifestyle, and behavioural factors^19–21,29–36^. In Canada, evolving building practices over the 20th to 21st century have increasingly and unintentionally captured, contained, and concentrated alpha radiation emitting radionuclides from radon within the residential built environment to unnaturally high and unsafe levels^29,31,37^. Radiation doses from radon inhalation are also a function of decision making factors and socioeconomics relating to radon awareness, testing, and reduction (i.e., how fast an individual is able to understand and reduce radon exposure as a personal risk factor)^20,21,30,38,39^, as well as lifestyle factors such as occupation that dictate activity patterns (i.e., the amount of time spent between environments)^19–21^. Based on current residential radon levels and activity patterns, radiation doses from radon in Canada are estimated to be at historic highs, with radon-vulnerable populations in Canada including younger people with children living at home, who are employed or in education, and/or whose occupations are more amenable to telecommuting^19^. In part, this phenomenon is driven by younger (those under age 45) Canadian adults being more likely to live in newer, more affordable residential buildings that have innately higher radon levels, and/or who are less able to afford radon reduction services due to comparatively lower household incomes^21^.

Socioeconomic disparities in lung cancer risk have been documented previously and are often linked to community types, with people living in more rural area (i.e. less populated) communities being more likely to use tobacco, earn less income, be exposed to different and/or higher amounts of environmental lung carcinogens throughout life, and have reduced access to health care and/or higher education^40–42^. In the context of radon exposure, disparities between urban and rural communities are documented and intriguing, but still ambiguous. Certainly, there are many reports of disparate rural versus urban indoor air radon levels across the globe^37,43–48^, highlighting a potentially serious issue with an ambiguous origin. One theory has been that domestic water supplies from rural-area groundwater wells often contain higher amounts of dissolved radon relative to (already de-gassed) supplies entering houses from urban-area municipal water treatment plants^49,50^. However, the contribution of water-borne radon to indoor air radon levels assessed in recent decades has typically been found to be relatively minor (~ 1–2% of total, equating to microsievert (μSv) doses)^51–57^; this is most likely because the equilibration ratio of radon from air to water is 1:10,000, meaning 10,000 Bq/L of radon degassing from water contributes only 1 Bq/m^3^ to air^49^. Thus, an important question becomes: if radon being released directly from groundwater to indoor air is not the biggest contributor to the phenomenon of higher rural radon exposure, as current research indicates, then what might it be?

How (and to what extent) radon exposure contributes to elevated lung cancer risks in diverse rural areas currently remains mechanistically unclear. In part, this is because earlier studies documenting radon differences between urban and rural areas were based on (i) smaller-scale datasets with limited statistical power (a few hundred radon readings), (ii) geogenic radon potentials (i.e., not empirical measurements of radon in indoor air), (iii) data without matched urban controls from the same region, (vi) data that were potentially confounded by uncontrolled differences in radon-modifying property metrics between the urban and rural built environment, and/or findings that could not connect individual exposures to lung cancer risk^37,52–57^. To address these knowledge gaps in a systematic manner, we surveyed indoor air radon levels in residential properties across a broad geographic region with a highly diverse urban to rural paradigm, controlling for differences in regional building metrics that influence radon, and deriving radon exposure outcomes, radiation doses, and increased cancer risks based on the activity patterns of people living in these diverse communities.

## Results

### Radon data collection and community type designations

**Figure 1:**
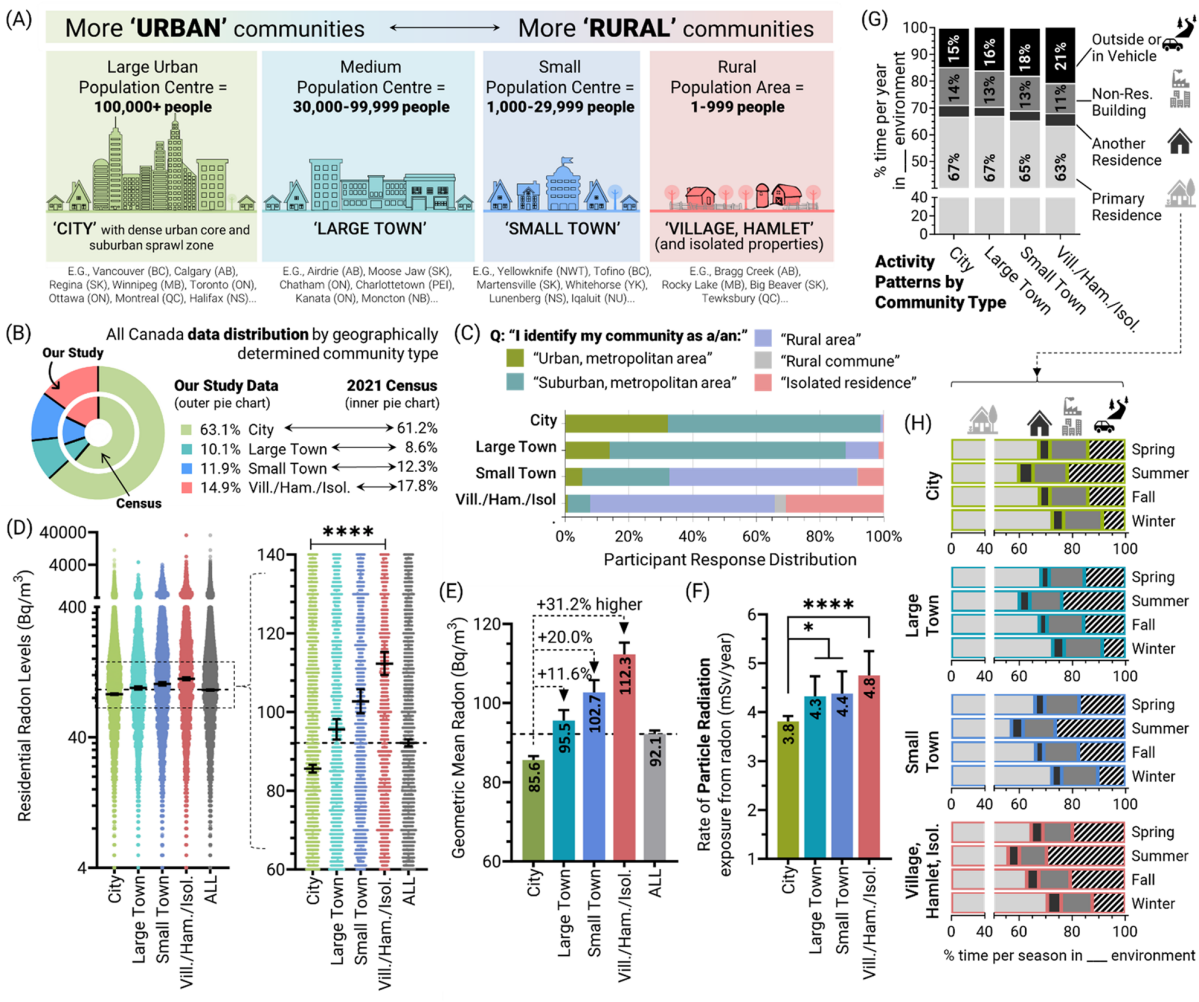
Rural community residential radon levels are higher relative to urban equivalents, resulting in excess radiation exposures. Panel (**A**) Schematic of the urban to rural community paradigm used within this study, including Canadian examples of population centre types. Panel (**B**) The distribution of residential buildings as a function of community types outlined in (**A**) for our study (outer pie chart) and the 2021 Canada Census (inner pie chart). Panel (**C**) Distribution of responses from occupants of the properties in our study after being asked to describe how they identified their community. Panel (**D**) Raw data for residential radon levels (n = 42,051) as a function of community type, with expanded region in right graph demonstrating geometric means. Dashed lines represent the geometric mean of whole dataset. Panel **E**. Geometric means from (**D**) indicating percent differences relative to city residential radon levels. Panel (**F**) Alpha particle irradiation dose rates (mSv/y) from radon in the primary residence of occupants across diverse community types. Panel (**G**) The percentage of time in a year spent occupying different environments including the primary residence (very light grey), a different residential building (medium grey), any non-residential building type (light grey), or in a vehicle or outside (black), as a function of community types. Panel (**H**) For each community type, the percentage of time per year spent in each environment (indicated by colours and icons used in (**A**) by season. Statistical analysis was done using ANOVA on log transformed data. **** = *p* < 0.0001; *** = *p* < 0.001; ** = *p* < 0.01; *p < 0.05; ns = *p* > 0.05. Figures were prepared using Excel and GraphPad Prism 9.1.1 (225) (www.graphpad.com). Other graphics were producing using Adobe Illustrator.

The study region included all Canadian provinces and territories, spanning a total land area of 9,984,670 km^2^ with (as of 2021) a population of 36.99 million people living in 16.28 million private dwellings. Based on Statistics Canada data, 17.8% of the Canadian population (6.6 million people in 2021) lived in a community of less than 1000 people (including isolated properties such as farm residences). Our study cohort encompassed 42,051 households who provided building, demographic, and radon test data to the ‘*Evict Radon National Study*’, a publicly funded project led by investigators at multiple Canadian universities^19–21,29–31,58^. All radon level outcomes were from alpha track style radon tests conducted for an average of 124.2 days and performed by citizen scientist participants with oversight of the study team. For the purposes of this work, all households were assigned a ‘community type’ identifier by combining the precise geographic information system (GIS) coordinates of the residential property tested for radon with contemporary Statistics Canada (StatCan) definitions of population centres. More specifically, the community types include large urban population centres (‘Cities’, ≥ 100,000 inhabitants), medium population centres (‘Large towns’, 30,000–99,999 inhabitants), small population centres (‘Small towns’, 1000–29,999 inhabitants), and entirely rural populations (“Villages, hamlets and isolated properties”, (1–999 inhabitants) (Fig. 1A). Based on these geographically derived designations, our cohort encompassed 2032 unique civic areas with 14.9% properties in villages, hamlets and isolated properties, 11.9% in small towns, 10.1% in large towns, and 63.1% in cities (Fig. 1B). Importantly, the distribution of communities in our cohort was highly symmetric with the outcomes of the 2021 StatCan national census, indicating that, overall, our sample of communities reflected Canada during the period of radon testing. To understand social perspectives of a civic area’s population relative to our GIS-associated classifications, we asked participants to report how they described the identity of the community in which their home was located. We found strong agreement between the perceptions of individuals about their community and the impartial designations produced by our team (Fig. 1C), supporting our classification system.

### Radon levels and related radiation exposures are higher in rural regions versus urban equivalents

We assessed average indoor air residential radon levels (i.e. alpha radiation emissions in Bq/m^3^) as a function of GIS-derived community types, and observed that properties in the most rural communities displayed significantly (*p* < 0.0001) greater geometric mean radon level (~ 112 Bq/m^3^), being up to ~ 31.2% higher on average compared to those in the most populous urban centres (~ 86 Bq/m^3^) (Fig. 1D–E). The upper 95% confidence interval (CI_95_) of radon in rural communities (CI_95_ = 529 Bq/m^3^ for small towns, 567 Bq/m^3^ for village-hamlet-isolated properties) was ≥ 200 Bq/m^3^ greater versus more urban equivalents (CI_95_ = 331 Bq/m^3^ for cities, 386 Bq/m^3^ for large towns), reflecting community-specific disparities even among houses deemed to have ‘high’ radon level outcomes. To better understand the consequences of these disparities, we estimated the absorbed radiation doses (to the lungs) from residential radon exposure per year, as a function of community type. To do this, we utilized our recently published human activity pattern data from ~ 4000 participants from across Canada^19^, and conversion formula from the *International Commission for Radiation Protection* (ICRP) to derive mSv/y dosimetry. In this context, activity patterns refer to the amount of time a person spends in their primary residence, versus at another residence, in a non-residential building, in a vehicle, or outside. Based on our GIS-derived community classifications, we found significant (*p* < 0.01) differences in radon-related absorbed radiation dose rates, being up to 1 mSv/y (~ 20%) greater for people living in villages, hamlets and isolated areas (annual absorbed radiation dose from radon = 4.8 mSv/y), relative to those living in cities (annual absorbed radiation dose from radon = 3.8 mSv/y) (Fig. 1F). Bearing in mind that indoor air radon levels are > 30% higher in rural versus urban communities, the reason there was ‘only’ a 20% excess in absorbed radiation dose from radon was that people in rural communities spent 4% less time per year in their primary home compared to those in cities, and 6% more time outdoors or in a vehicle (Fig. 1G). The trend of reduced rural occupancy within the primary residence remained consistent across seasons (Fig. 1H) and explains the partially attenuated impact of higher radon levels in rural communities on absorbed radiation doses.

### Higher rural residential radon levels are universal across Canada, irrespective of region

**Figure 2:**
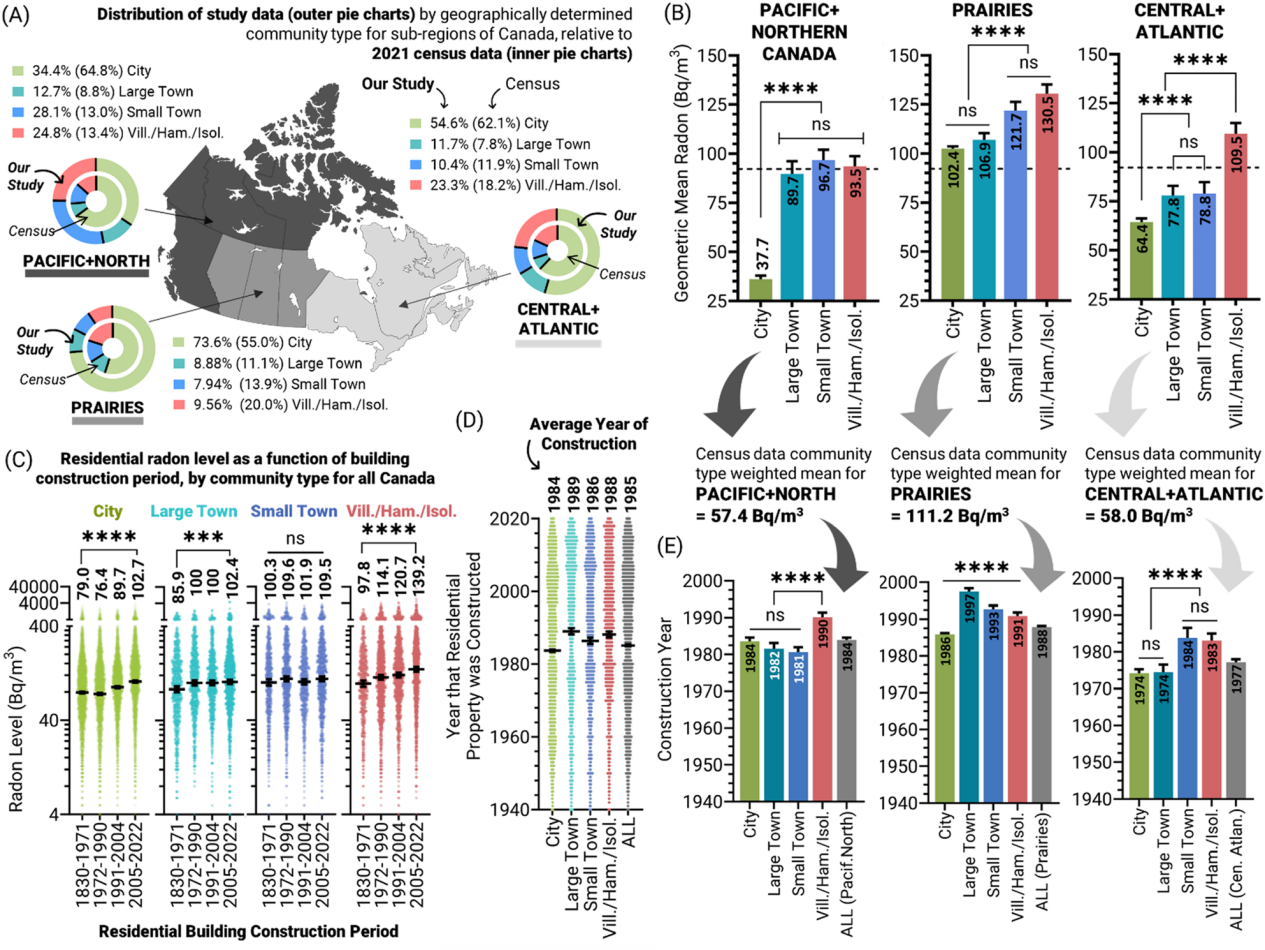
Urban-to-rural community radon level and year of construction trends by sub-region. Panel (**A**) A map of three indicated sub-regions of Canada, showing the distribution of residential buildings as a function of community types for our study (outer pie charts) and the 2021 Canada Census (inner pie charts, and percentage values within brackets). Panel (**B**) Geometric mean residential radon levels by community type for each Canadian sub-region outlined in (**A**), with percentages from the 2021 Canada Census used to re-weight data to derive geometric means for each sub-region. Panel (**C**) Residential radon levels by building construction period quartiles. Panel (**D**) Raw data and geometric mean year of construction for each community type for all of Canada. Panel (**E**) Geometric mean year of construction for residential properties in each community type for the three Canadian sub-regions described in Panel (**A**) Statistical analysis was done using ANOVA on log transformed data. **** = *p* < 0.0001; *** = *p* < 0.001; ** = *p* < 0.01; *p < 0.05; ns = *p* > 0.05. Figures were prepared using Excel and GraphPad Prism 9.1.1 (225) (www.graphpad.com). Maps were produced in ArcGIS Pro 3.1.2.

We next explored whether our observations were consistent across Canada, separating our cohort into three aggregate regions including (i) Pacific and Northern Canada (British Columbia, Yukon, Northwest Territories, and Nunavut), (ii) Prairie Canada (Alberta, Saskatchewan, Manitoba), and (iii) Central and Atlantic Canada (Ontario, Quebec, New Brunswick, Nova Scotia, Prince Edward Island and Newfoundland) as we did recently^31^ (Fig. 2A). This sub-division revealed some asymmetry between our study and the 2021 census in terms of community type distribution, with the greatest differences being an over-representation of Prairie cities, rural Pacific-North and Central-Atlantic areas, and an under-representation of Pacific-Northern and Central-Atlantic cities. To account for this, we ascertained the geometric means for all community types for each sub-region, and weighted this using census data for community type distribution (Fig. 2B). As observed previously^31,37^, the overall geometric mean radon for residential properties was highest in the Prairies (111.2 Bq/m^3^), followed by Central and Atlantic Canada (58.0 Bq/m^3^), and then Pacific and Northern region (57.4 Bq/m^3^) (Fig. 2B).

In all sub-regions, rural communities showed significantly (*p* < 0.0001) greater radon levels relative to properties in matched urban areas. Given the diversity of the underlying geologies, climates, and building portfolios (housing ages, designs, etc.) across Canada, the consistency of the higher rural radon effect was striking, reflects trends observed internationally^37,52–57^, and suggests a common cause.

### Accounting for construction period differences between urban and rural communities

We next investigated the mechanism underlying the higher rural radon phenomena. One of the most influential predictors of high residential radon exposure in Canada is construction period, with more recently built residential properties containing substantially higher radon relative to older properties^21,29,31,37^. To account for potentially confounding differences in property ages between community types, we separated our data by construction period quartiles. In general, more recently constructed properties contained greater radon levels versus older buildings, particularly those built after 2005 relative to pre-1972 equivalents (Fig. 2C). Trends in year of construction for a given community (Fig. 2D–E) did not correlate with the pattern of differences in radon levels, either across Canada, or within a sub-region (Fig. 1E, 2B–C). Overall, we conclude that the relatively small differences in construction periods could not adequately explain community type differences in radon exposure.

### Accounting for floorplan size differences between urban and rural communities

**Figure 3:**
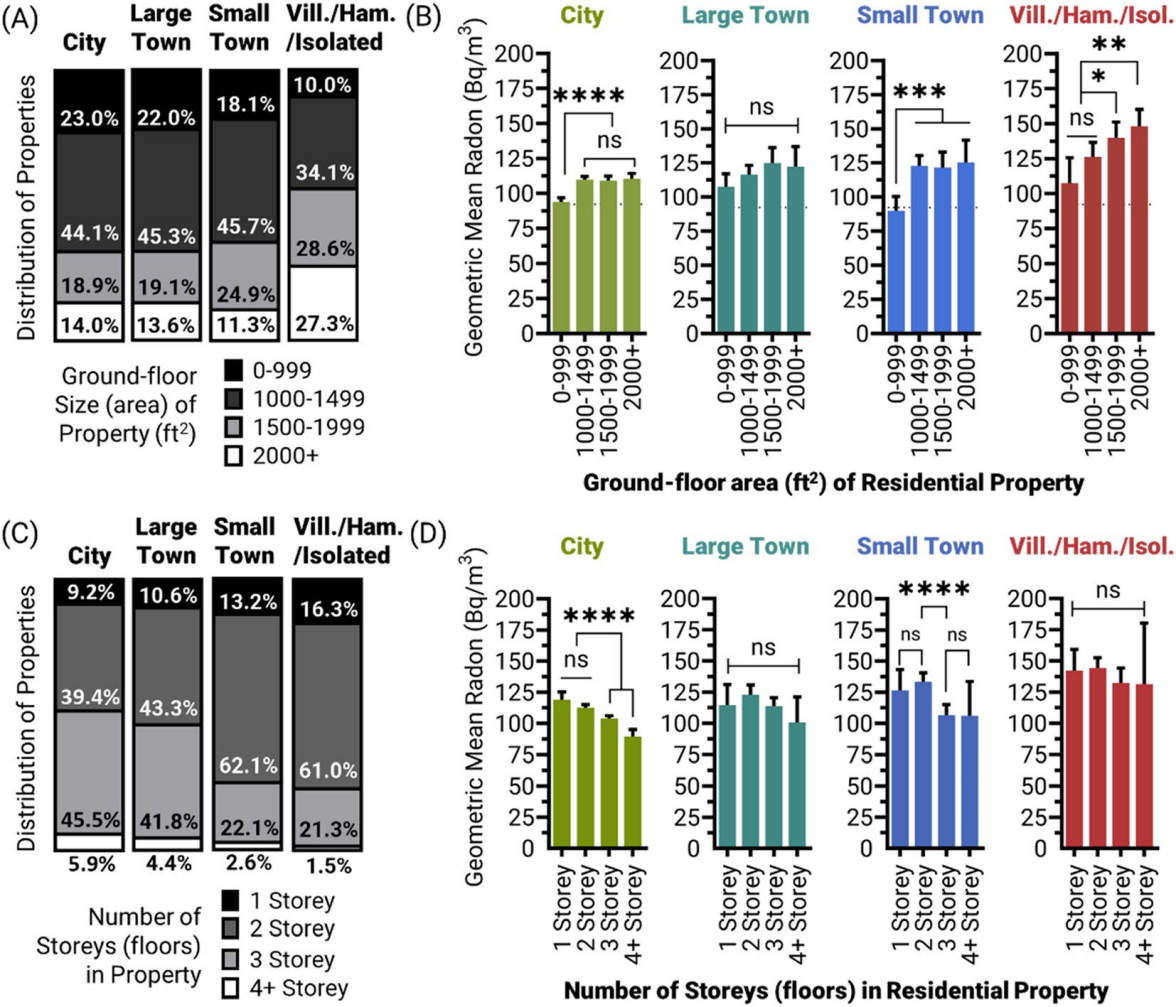
Urban-to-rural residential radon levels as a function of ground floor size and number of floors in the property. Panel (**A**) Distribution of residential properties by ground floor size (area, reported in ft^2^) for each community type. Panel (**B**) Geometric mean residential radon levels as a function of ground floor size groups, for each community type. Panel (**C**) Distribution of residential properties by total number of floors (reported in number of storeys, i.e., habitable levels to the property) for each community type. Panel (**D**) Geometric mean residential radon levels as a function of total number of floors, for each community type. Statistical analysis was done using ANOVA on log transformed data. **** = *p* < 0.0001; *** = *p* < 0.001; ** = *p* < 0.01; *p < 0.05; ns = *p* > 0.05. Figures were prepared using Excel and GraphPad Prism 9.1.1 (225) (www.graphpad.com).

Another major predictor of higher residential radon in Canada is ground floor size (surface area), with larger properties displaying higher radon levels compared to smaller properties^29^. A comparison of ground-level floor sizes (reported by participants in square feet, ft^2^) indicated large differences between community types, with village, hamlet and isolated areas reporting substantially more ‘very large’ (> 2000 ft^2^ ≅  > 185 m^2^) and half as many ‘very small’ (< 1000 ft^2^ ≅  < 92.9 m^2^) residential properties versus any other community type (Fig. 3A). In most communities, larger properties corresponded significantly (*p* < 0.05–0.0001) with greater indoor air radon levels (Fig. 3B). However, the magnitude of the radon-increasing effect (associated with property size) was more than doubled in small towns and village, hamlet and isolated communities (a 37–44% increase in radon between smallest to largest houses) compared to cities (a 17–18% increase in radon between smallest to largest houses). These data suggest that the radon-amplifying effect of a larger property footprint (i.e., the area in contact with the ground) is greater in rural areas relative to their urban equivalents.

### Accounting for floor (storey) number differences between urban and rural communities

Another building feature that influences radon levels in Canadian residential properties is the overall number of floors of livable space (storeys), with greater storey number equating to lower radon^29,37^. In this case, a fully or partially below-grade basement is included within the storey count, while a crawlspace is not. The impact of greater storey number on reducing radon is thought to be a dilution effect associated with the extra windows and doors that follow with each additional floor. As with residential building floorplan size, we also observed large differences in storey number between community types, with 46–52% of properties having ≥ 3 storeys in cities and large towns, versus < 25% in rural communities (Fig. 3C). Curiously, while increased storey number correlated significantly (*p* < 0.0001) with lower radon in cities, there was no significant (*p* > 0.05) trend observed for villages, hamlets and isolated areas (Fig. 3D). Indeed, ≥ 3 storey buildings in villages, hamlets and isolated areas showed comparably high radon to ≤ 2 storey houses in the same community type, and much higher radon versus their more urban equivalents. Altogether, this suggests that the radon-suppressing effect of greater storey number is absent or negated some community types, including rural areas.

### Accounting for building design differences between communities

**Figure 4:**
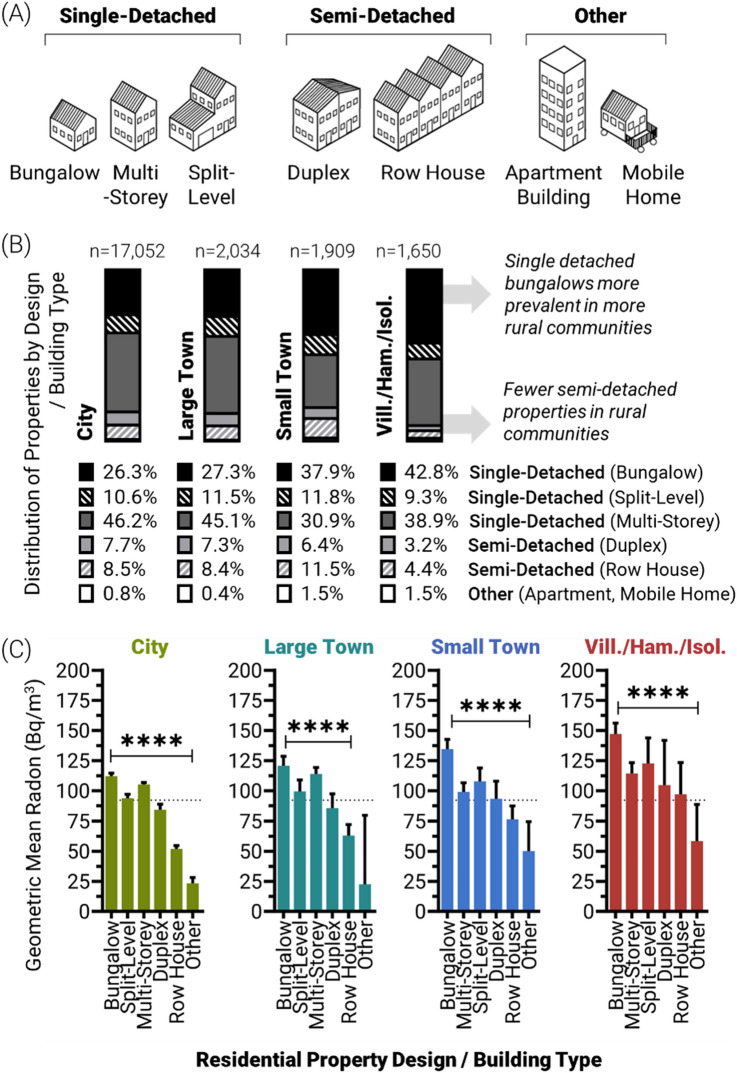
Urban-to-rural residential radon levels as a function of building design type. Panel (**A**) A schematic of the six categories of building design type considered within this study. Panel (**B**) Distribution of residential properties by building design type. Panel (**C**) Geometric mean residential radon levels as a function of building design, for each community type. Statistical analysis was done using ANOVA on log transformed data. **** = *p* < 0.0001; *** = *p* < 0.001; ** = *p* < 0.01; *p < 0.05; ns = *p* > 0.05. Figures were prepared using Excel and GraphPad Prism 9.1.1 (225) (www.graphpad.com). Other graphics were producing using Adobe Illustrator.

In terms of building design type, we found previously that bungalow style, single-detached properties contained the highest radon, while semi-detached row style and/or multifamily dwellings contained the lowest^29,31^. Across Canadian community types, we observed noticeable differences in the distribution of building design types, with single detached properties (and bungalows in particular) being the most prevalent in low population communities (Fig. 4A–B). Irrespective of building type, rural properties contained greater radon versus urban equivalents (Fig. 4C); however, as expected, single-detached houses contained higher radon versus semi-detached houses, with bungalows being highest in all community types. Given the greater prevalence of single detached bungalows in more rural areas observed in Fig. 4B, this raises the possibility that the increased prevalence of higher radon single detached properties in rural communities might underlie a proportion of the excess radon levels in these areas.

### Accounting for the presence of groundwater wells between community types

**Figure 5:**
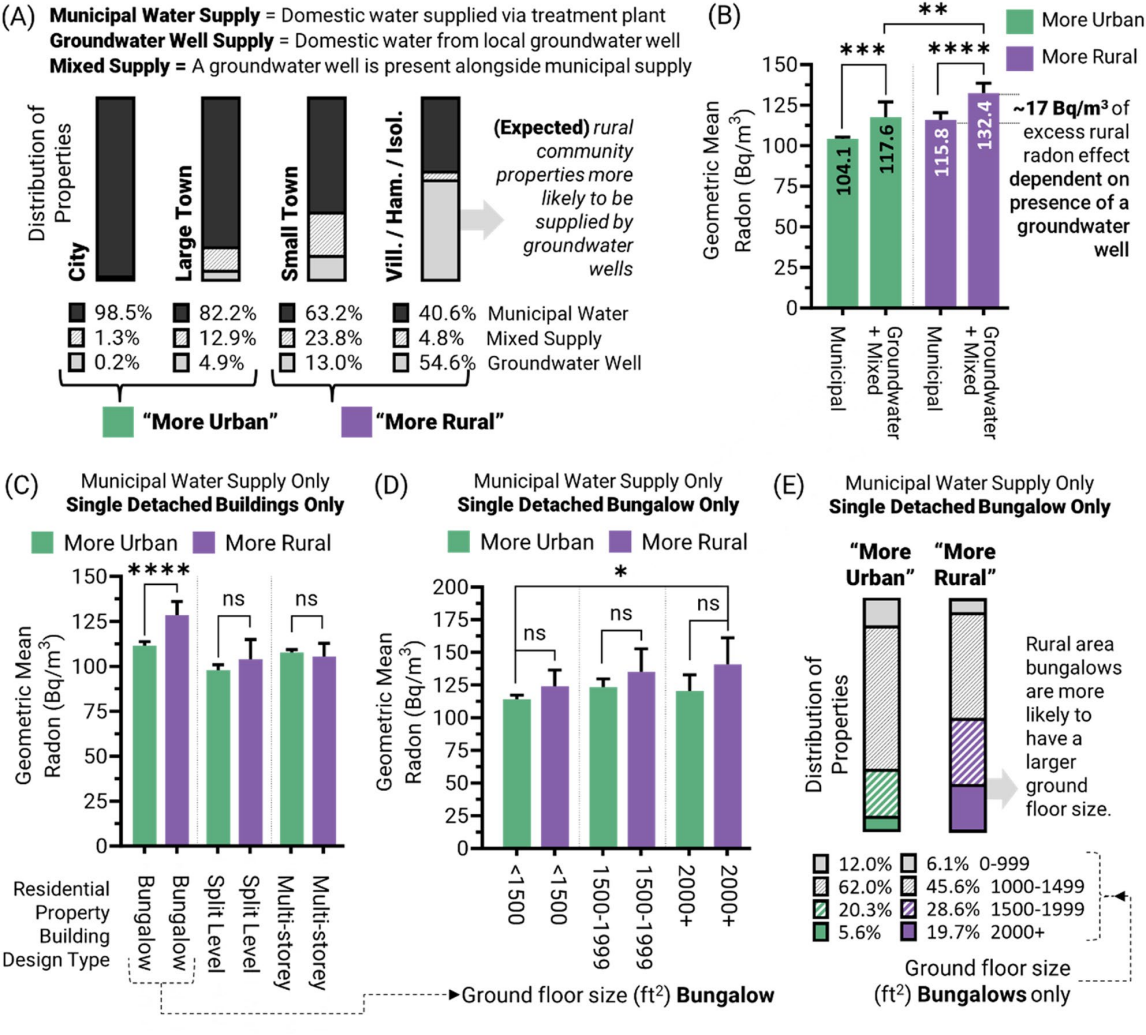
Groundwater well supply prevalence corresponds to a majority of the urban-to-rural disparity in residential radon exposure. Panel (**A**) Distribution of residential properties according to water supply (municipal, groundwater well, or both). Panel (**B**) shows geometric mean residential radon levels as a function of domestic water supply source, for ‘urban’ (large and small cities; teal green) and ‘rural’ (small town, villages, and isolated; purple) community types. Panel (**C**) For properties on municipal water only, geometric mean residential radon levels as a function of single detached houses design type. Panel (**D**) Distribution of bungalows on municipal water by ground floor size for urban and rural communities. Panel (**E**) For bungalows on municipal water, geometric mean residential radon levels as a function of ground floor size. Statistical analysis was done using ANOVA on log transformed data. **** = *p* < 0.0001; *** = *p* < 0.001; ** = *p* < 0.01; *p < 0.05; ns = *p* > 0.05. Figures were prepared using Excel and GraphPad Prism 9.1.1 (225) (www.graphpad.com).

We next examined if the presence of groundwater wells—a well known difference between urban and rural areas—correlated with indoor air radon differences between communities. Participants reported whether their household water supply was from either municipal water, a groundwater well, or mixed supplies (i.e., both groundwater and municipal sources). As expected, there were very few houses in urban areas that relied on groundwater wells, with the reverse being true in rural areas (Fig. 5A). Indeed, 98.5% of city households were supplied by municipal water, while in villages, hamlets, and isolated properties ~ 60% of water was obtained entirely or in part from groundwater, with large and small towns showing intermediate distributions. To overcome (from a statistical analysis standpoint) the relatively small number of residences with groundwater as a water source in cities and large towns, we grouped these communities together as “urban” and compared them to a combination of small towns, villages, hamlets, and isolated properties as ‘rural’. Importantly, for residential properties supplied by a groundwater well, there was no statistical difference (*p* > 0.05) in geometric mean radon concentrations between urban and rural communities, with the presence of groundwater wells corresponding with up to + 17 Bq/m^3^ excess radon levels, and accounting for a majority of the excess rural radon effect (Fig. 5B) observed for properties reporting their water source.

### Accounting for groundwater well-independent urban–rural differences in radon

We considered whether urban and rural differences in radon might be a function of parameters other than groundwater well presence, for example variable building design types and/or floorplan sizes (as outlined in Figs. 3, 4). To examine this, we isolated single detached houses without a groundwater well, and found that only bungalows displayed significant (*p* < 0.0001) urban versus rural differences (Fig. 5C). However, even these differences were erased when the same buildings were analyzed as a function of ground floor size and, although larger floor plan properties still had significantly (*p* < 0.001) greater radon in general, there were no significant (*p* > 0.05) urban to rural differences observed if floorplan differences were accounted for (Fig. 5D). As 48.3% of rural area bungalows are > 1500 ft^2^ in size, versus only 25.9% in urban communities (Fig. 5E), the increased prevalence of the larger bungalows in rural areas (which contain higher radon, Fig. 3B) is a strong explanation for groundwater well independent differences in radon between urban and rural Canadian communities. Overall, these outcomes suggest that part of the difference in residential radon between urban and rural Canadian communities is because rural residential properties are more likely to be larger, bungalow-style buildings, while a majority of the effect is due to the presence of groundwater wells. As groundwater wells are a fundamental, prevalent feature of the rural built environment across most of the world, this factor might explain why rural versus urban disparities in radon exposure have been observed in so many diverse regions.

### Canadian groundwater radon levels are too low to account for excess rural radon trends

**Figure 6:**
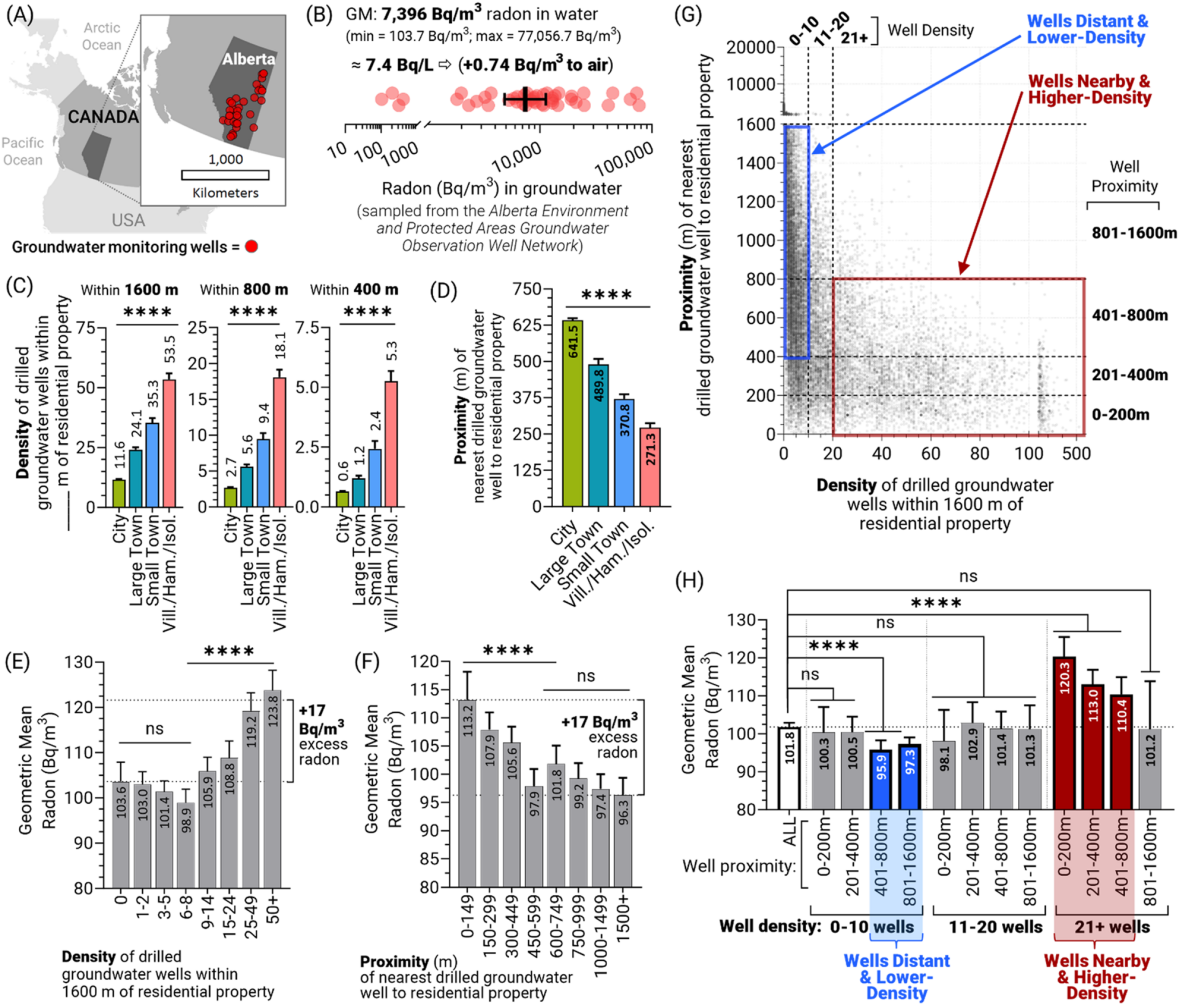
The relationship between drilled groundwater well proximity, well density, and well water supply to residential property indoor air radon levels. Panel (**A**) Map showing the Canadian province of Alberta, and the location (red dots) of groundwater monitoring wells withing the Alberta Environment and Protected Areas Groundwater Observation Well Network that were sampled. Panel (**B**) Radon concentrations (Bq/m^3^) in groundwater sampled from the locations indicated in (**A**), showing geometric mean (GM), conversion to Bq/L and estimated contribution to indoor air. Panel (**C**) The geometric mean density of drilled groundwater wells within a 1600 m, 800 m, or 400 m radius of residential properties for all community types. Panel (**D**) The geometric mean of the proximity (m) of the nearest drilled groundwater well to residential properties across community types. Panel (**E**) Indoor air residential radon levels as a function of groundwater well density within a 1600m radius of the property. Panel (**F**) Indoor air residential radon levels as a function of nearest groundwater well density proximity (m) to the property. Panel (**G**). Drilled groundwater well proximity (y-axis) versus density (x-axis) for all households in our cohort. The blue box highlights residential properties with more distant, AND a lower density of drilled groundwater wells. The red box highlights residential properties with nearby AND a higher density of drilled groundwater wells. Panel (**H**) Geometric mean radon levels within residential property indoor air, using the highlighted groups for drilled groundwater well density and proximity shown in (**G**). Statistical analysis was done using ANOVA on log transformed data. **** = *p* < 0.0001; *** = *p* < 0.001; ** = *p* < 0.01; *p < 0.05; ns = *p* > 0.05. Figures were prepared using Excel and GraphPad Prism 9.1.1 (225) (www.graphpad.com). Maps were produced in ArcGIS Pro 3.1.2.

As outlined earlier, current models suggest that approximately 10,000 Bq/m^3^ of radon in water (which is equivalent to 10 Bq/L in water) is needed to increase radon levels in air by 1 Bq/m^3^; this implies that to observe a + 17 Bq/m^3^ average excess of radon in indoor air attributable entirely to radon degassing from groundwater, that average regional radon levels in groundwater would need to be at least 170,000 Bq/m^3^ (170 Bq/L). Using Alberta Environment and Protected Area’s *Groundwater Observation Well Network*, a provincial government owned series of monitoring wells in various aquifers, we monitored dissolved radon concentrations in drilled groundwater wells in forty rural community regions (Fig. 6A). While there was substantial variation (min = 100 Bq/m^3^; max = 77,000 Bq/m^3^), most groundwater well water radon levels clustered around a mean of 7400 Bq/m^3^ or 7.4 Bq/L (Fig. 6B). When considered together with radon levels in groundwater documented over the past decade using similar techniques in the Canadian provinces of Nova Scotia (5.7 and 8.1 Bq/L)^59,60^ and Quebec (8.6 Bq/L), as well as large studies conducted in the USA (9.2 Bq/L), and China (11.8 Bq/L)^61–63^, this suggests that Canadian groundwater radon levels are comparable to those globally, but that they are also an order of magnitude below the amounts needed to account for excess rural radon trends. More specifically, and assuming no loss in dissolved radon between well heads and residential building taps (which is unlikely), the mean value of 7.4 Bq/L dissolved in Albertan groundwater would equate to ~ 0.74 Bq/m^3^ to average indoor air levels if all of the dissolved radon in well water were degassed—i.e., only a tiny fraction of the + 17 Bq/m^3^ average excess associated with the presence of a groundwater well (Fig. 6B). From another perspective, a widely used transfer coefficient for radon in indoor air would be expected to result in a radon concentration increase of 0.74 Bq/m^3^ in indoor air^64^.

### Groundwater well density and proximity to property as high indoor air radon risk factors

If, as our data suggests, > 95% of excess radon levels observed in residential buildings supplied by groundwater is unlikely to be from radon degassing from water itself, then there must be feature(s) in rural residences that form different radon pathways compared to urban homes. A major difference between urban and rural home construction relates to household water supply and disposal.

While urban homes will have shallow (i.e., < 4 m depth) infrastructure that pipes water into, and wastewater out of, residences, most rural residences depend on water wells for water supply and onsite wastewater treatment systems for disposal. Onsite wastewater treatment system (or ‘septic system’) infrastructure tends to be shallow (i.e., similar to the depth of urban water and wastewater infrastructure). Rural water wells tend to be orders of magnitude deeper, however, than urban infrastructure, ranging typically from a few meters (for hand dug wells) to a several hundred meters of depth^65,66^. Generally, water wells in Canada are drilled to the depth at which the water well driller ascertains that groundwater can be pumped out of the well at a sufficient rate for the required water household supply. Based on public records, water well depths in the Canadian province of Alberta are an arithmetic average of 139 m (median = 40 m) deep, based on a sample size of 419,097 wells^67^. Both the water well depth and completion, i.e., the depth to which the well screen and tubing (known as ‘casing’) is inserted into the well, and how the well is ‘sealed’ to prevent unintended fluid migration pathways are variable.

The space between a drilled borehole and well casing, referred to as the ‘borehole annulus’, has been recognized as a significant pathway for methane gas migration around oil and gas wells, irrespective of regulations that mandate more stringent seal placement of an oil and gas well annulus compared to water wells^68–71^. In the Canadian province of Alberta, for example, current regulations require cement seals be placed in the borehole annulus of oil and gas wells to at least 180 m depth^72^. While cement is used because it has relatively low permeability, it stills permit buoyant free phase gas migration in the well annulus^70,71^ along vertical pathways that can be hundreds of metres deep^73^. Given that water well installation and completion regulations are much less stringent than those for oil and gas wells, their borehole annuli likely provide even better free phase gas pathways for gaseous, buoyant migration of radon-rich gas in groundwater zones. Based on this knowledge, we hypothesize that radon in rural community houses is relatively higher because radon-rich gas migration (from deeper geologic sources) occurs favorably in the annulus of drilled groundwater wells. The potentially very fast (i.e., km/day rates)^74,75^ rates of buoyant free phase gas migration in the subsurface can permit locally higher radon concentrations from depth (i.e. orders of magnitude greater than those observed in residences)^76^ to reach the near subsurface, from whence radon diffuses upwards and outwards to surficial soil layers to generate an innately higher radon concentration in soil gas^76^. Rural residences could capture and retain greater amounts of alpha radiation emitting radionuclides (i.e. radon and its decay products) from these radon-enriched, near-surface soil gasses, an idea supported by the observation that the radon-increasing effect associated with property size (i.e., the area in contact with the ground) was greater in rural versus urban communities (Fig. 4A–B).

To test the ideas outlined above, we explored the correlation between indoor air radon and groundwater well density and water well proximity (to residential properties) using the Canadian province of Alberta, as this is a study region where we had access to locations of drilled groundwater wells that were ≤ 1600 m. The density of drilled groundwater wells within radii between 400 and 1600 m was calculated for each residence in our cohort, with a 5- to tenfold difference in groundwater well density observed between urban-to-rural communities. Rural villages, hamlets and isolated areas had an average of 5.3 wells within a 400 m radius, and > 50 within 1.6 km compared to > 1 for cities (Fig. 6C). More rural community area properties were also more proximal to groundwater wells (Fig. 6D). Indoor residential radon levels showed a significant (*p* < 0.0001), positive correlation to both increased drilled groundwater well density and proximity, with the lowest to highest extremes between each showing a + 17 Bq/m^3^ difference in radon (Fig. 6E–F). We note that this difference was comparable in magnitude to the overall excess radon observed as a function of groundwater usage shown in Fig. 5B. When examined together, it was clear the drilled groundwater well proximity and density were partially co-dependent variables (Fig. 6G), although there were some significant (*p* < 0.001) additive effects displayed by houses with very nearby and high-density wells (highest radon) versus very distant and low-density wells (lowest radon) (Fig. 6H). Collectively, these data support the idea that the presence of drilled groundwater wells (as opposed to the degassing of radon from water pumped from these wells) accounts for a majority of the excess rural radon effect observed across Canadian communities.

## Discussion

**Figure 7:**
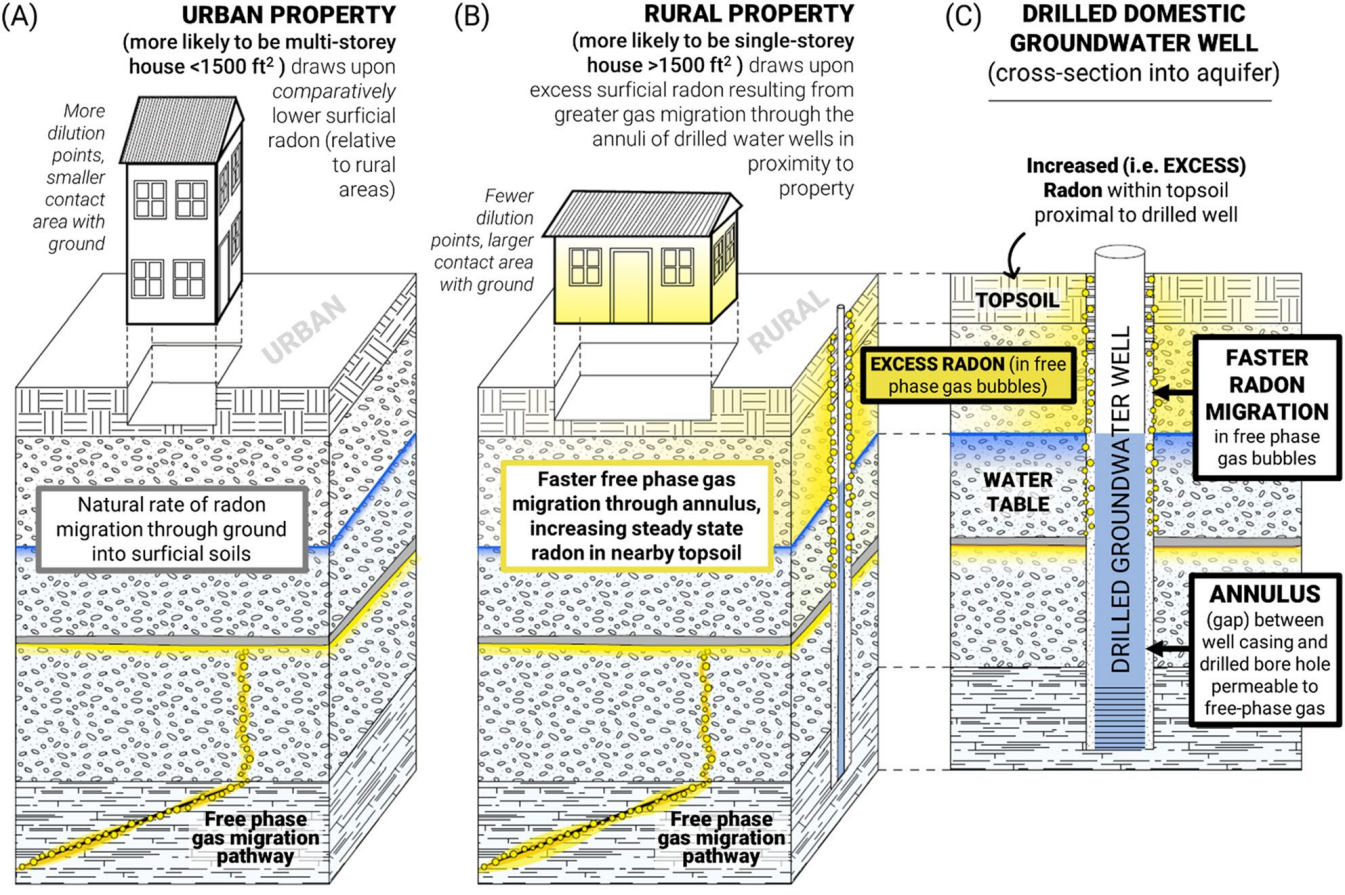
The comparatively higher indoor air radon levels in rural community residential houses due to property design type differences and excess surficial radon arising from drilled well effects. Panel (**A**) A schematic representing urban community residential properties whose indoor air radon levels are a function of the prevalence of multi-storey housing (< 1500 ft^2^ in size), as well as the natural rate of free phase gas migration through ground into surficial soil. Panel (**B**) A schematic representing rural community residential properties whose indoor air radon levels are a function of the prevalence of single-storey housing (> 1500 ft^2^ in size), as well as increased radon levels in surficial soil due to an accelerated rate of migration of free phase gas through the annuli of nearby drilled water wells. Panel (**C**) An expanded cross-section of a drilled domestic water well located nearby a typical rural community residential property, showing excess surficial radon (yellow shading) in upper ground layers arising from free phase gas bubbles (yellow circles) moving more readily through the water table (indicated by blue line) and ground via the well annulus (the gap between well casing and bore hole). Graphics produced using Adobe Illustrator.

In this study, we document a major disparity in radon exposure across the urban to rural community paradigm, with people living in more rural communities experiencing substantially greater indoor air radon levels in a manner largely independent of geography. Our data raises the possibility that much of the excess rural radon effect could be attributable to increased free phase gas migration (containing radon) through the annulus of drilled domestic water wells (Fig. 7). The remaining (smaller) radon level differences are accounted for by differing trends in house design and sizes between communities. We emphasize that the drilled water wells do not contribute to indoor air radon via pumping well water into buildings, as documented levels of dissolved radon groundwater are not present in sufficient quantities to produce observed differences between community sizes. Rather, we speculate that unintended radon gas migration along preferential pathways formed along the annuli of drilled groundwater wells (i.e., the free phase gas-permeable gaps between boreholes and well casings) effectively transports high fluxes of gaseous radon through the groundwater zone and into the unsaturated zone above the water table, causing elevated radon concentrations in shallow soil below and migration into residential properties by ‘vapour intrusion’^77^. We stress that this is a completely unintended phenomenon that would be very difficult to avoid for properties whose only option for water supply are drilled wells. Water well completion regulations (under provincial and territorial jurisdiction in Canada) have varying degrees of annular seal requirements, and (in public record and to our knowledge) have historically not been designed to mitigate the impact of gas migration around wells on indoor air radon. Importantly, for impacted communities, the existing solutions to a high indoor air radon problem (i.e., a sub-slab depressurization radon reduction system) are likely to elicit greater radon reductions and be a more cost-effective solution than well intervention^78^.

The higher rural residential radon trends that we have observed are consistent across the very diverse geology, climate, and built environment of Canada (the world’s second largest nation by land area). As such, we suggest that the trends we have observed in Canada may also apply internationally, perhaps especially in countries with comparable groundwater radon levels, such as China and USA^37,43–48^. From a lung cancer risk perspective, our data suggest that higher rural radon levels do translate to increased radon-emitted alpha particle radiation doses to the lungs. The increased radiation doses that rural community populations experience from radon inhalation are 20% greater on average than people living in cities based on the data we show in Fig. 1F, and would be higher but for people in rural Canadian communities spending comparatively less time per year in their primary home (and more time outside or in a vehicle). Understanding this widespread disparity is important since nearly one fifth of the Canadian population live in a rural community: a very large group of people who are already known to be at differential risk of lung cancer due to a comparatively greater rate of tobacco smoking, reduced access to healthcare, and distinct exposures to other environmental lung carcinogens (such as arsenic in water, combustion particulates from vehicles, etc.)^21,36,41,42,79–82^. In terms of policy recommendations, this work makes a clear public health argument to direct resources to rural communities for radon awareness, testing, and reduction services.

In general, the terms ‘urban’ and ‘rural’ are concepts that are understood intuitively by most people, and reflect relative differences in population densities across a continuum. In this paradigm, it is important to acknowledge that how people conceptualize their community identity is a product of their personal and shared experiences and needs, as well as built environments. Community classifications can also change with time, particularly in North America where the ongoing periurban expansion of larger population centres can absorb neighbouring areas to produce mixed community identities and/or built environment with a legacy of rural infrastructure (such as groundwater wells) within a newer urban framework^83^. A positive outcome from our current study is that, with a specific mechanistic explanation for the differences in indoor air radon between urban and rural communities (i.e., higher rural residential drilled groundwater well proximity and density, and greater prevalence of larger single-storey properties), policy makers, health providers, and knowledge translators have an actionable target for risk evaluation and intervention that is more specific than simply ‘rural’. As just one example, we predict that knowing that a person has lived within a property with a (likely) high density of nearby groundwater wells may prove useful as part of eligibility criteria for lung cancer screening beyond current methods that primarily consider tobacco smoking, family cancer history, and highest education (a readout of socioeconomic status). As this risk metric can be estimated based on property address without the occupant needing to have specific knowledge, it may prove advantageous to risk assessment.

In terms of strengths and weaknesses, one strength of this work is that it rests upon a robust understanding of > 42,000 long-term indoor air radon readings from across Canada. To our knowledge, this is one of the first Canadian studies on residential radon to compare the effect of community type. The outcomes of this work highlight the need to contextualize radon exposure as a multifaceted issue that spans building and behavioural science to population centre characteristics. Comparison to the most recent (2021) Canada census reveals a generally strong symmetry to community type distributions in Canada as a whole and, through region-specific analysis, we have identified asymmetries and have accounted for that wherever possible. We acknowledge one limitation is that, for our analysis of the relationship between radon and groundwater well density and proximity, we focused on one case region (the province of Alberta) for practical reasons, and that it would be beneficial to expand this more broadly across Canada in future work.

Monitoring the near subsurface (i.e., soil gas radon) for gas migration^73^ in locations with a higher density of drilled water wells and/or very high indoor radon concentrations relative to control (e.g., few to zero well) areas will help consolidate our hypotheses. In the near future, we also think it important to directly assess the cumulative impact of all drilled wells and natural geologic structures (e.g., faults, fractures) on residential radon levels, including fossil fuel, wastewater injection, and geothermal wells. While we might anticipate that the annuli of such non-domestic wells may have a similar effect to drilled domestic water wells on radon migration, this is likely confounded for two reasons. First, the prevalence of other well types across communities in Canada (and globally) is inconsistent (i.e., they have been disallowed in four of ten Canadian provinces for more than a decade). Second, while energy wells are typically drilled much deeper than water wells, they are more tightly regulated in how annuli are sealed, potentially attenuating their impact on free phase gas migration; this is in contrast to water wells, for which information about well annuli seals are often not available^84^. Similarly, natural geologic structures (e.g. fractures, faults) could provide effective gaseous radon transport pathways. Future work would be required to account for the combined, relative impact of non-domestic groundwater wells, and natural geologic structures.

Finally, the outcomes of our work support the conclusions from multiple, previous studies that suggest the contribution of radon from water supplied from drilled groundwater wells is not a significant contributor to indoor air radon in a majority of cases. While this does not preclude health concerns regarding direct consumption of water with high dissolved radon concentrations, it does shift the emphasis away from radon degassing from domestic groundwater as a primary driver of high radon within indoor air, and agrees with current advice from governments and other advisory bodies. In closing, we emphasize that people living in more rural areas of Canada typically are of reduced socioeconomic means compared to more urban populations, and experience reduced access to radon reduction services in general. As such, a major recommendation of this study is for authorities to direct resources to rural communities to help resolve this environmental cancer risk disparity.

## Methods

### Statement of approvals

All activities were pre-approved by the Conjoint Health Research Ethics Board, Research Services, University of Calgary (IDs = REB17-2239, REB19-1522) adhering to citizen science research best practice^58^, and in accordance with all regional guidelines and regulations. Work was peer-reviewed in advance and funded by four public sector agencies: Health Canada, the Canadian Institutes for Health Research, the Alberta Innovates Water Innovation Program, and the Alberta Real Estate Foundation. The research team was head-quartered across three separate units (Medicine, Architecture, Earth, Energy and Environment) at the University of Calgary, and involved the staff of the Evict Radon National Study.

### Enrollment and surveying

All participants active within the Evict Radon National Study as of 2023 were invited to complete online surveys and questionnaires using the Qualtrics survey platform. Study enrollment for this study was based on convenience recruitment for all wanting to join, with all adult homeowners and renters in any residential building type being eligible. Enrollment opportunities were communicated broadly, and across the study region via multiple platforms including online and mass media via professional journalism. No data from any constituent part of this cohort were from known or pre-selected lung cancer cases. Wherever possible, data was expressed as a function of single or combined demographics to contextualize outcomes. In addition to the detailed analyses presented in this study, many of the human demographics and building metrics associated of this cohort have been previously peer-reviewed and described in^20,21,29–31^. To summarize the major demographic features of the cohort reported in those studies for convenience, our participants gross (before tax) household income is comparable to the average household income of Canada at the time that testing occurred (CAD$118k/y), with 56% reporting an income between $60–200k, and 27% an income of less than CAD$60k/y, and 17% an income of more than CAD$200k/y. The mean age of participants was 53.9 years of age. In terms of gender identity, the cohort was 40% cisgender women, 48% cisgender men, 0.1% gender minority, with 12% declining to report. The cohort were gender-balanced between ages 18 to 65, with a small skew in favour of people identifying as men for the over 65 age group. A majority (59%) of our cohort report being in full or part time work at the time of radon testing, with 35.5% being retirees, and the remainder being either on long term leave or unemployed. Records of informed consent were obtained in all cases, and participants were permitted to withdraw at any time. All identifying information was removed early on during data analysis, and absolutely no identifying information is included within the data presented in this study for publication. A list of survey questions relevant to this study is outlined in “Supplemental Information”.

### Radon testing

From 2015 to 2023, Canadians enrolled in the Evict Radon National Study purchased RadTrak2 (2013–2020) or RadTrak3 (from > 2020) alpha track 90 + day radon detectors at cost, that were then shipped directly to their home. Participants then deployed tests for a > 90 days, returned them for analysis, and later received their specific radon reading in a confidential manner. Non-profit study kits ranged from CAD$45–53, depending on year (price differences driven by inflation and material costs across 2015–2023). Radon outcomes and population-based characteristics of this cohort have been described extensively and recently in^19–21,31^.

### Radiation exposure calculations

To convert Bq/m^3^ indoor air radon levels to human mSv radiation exposures (to lungs), the ICRP provides the following formula^85^:

$$\left( {6.7 \times 10^{ - 6} mSv\,per\,Bq \cdot h/m^{3} } \right) \times \left( {\left[ {radon} \right]Bq/m^{3} } \right) \times \left( {\left[ {time\,in\,residence\,per\,year} \right] \, h/y} \right) = mSv/y$$

Values for the amount of time spent in the primary residence per year for a typical adult (*“time in residence per year”)* were calculated from individually reported residential occupancy data from 4009 participants, and cross-referenced with data from the *National Human Activity Pattern Study* (NHAPS)^86^ and more recent Canada-specific updates^19^. Participants reported activity pattern data by season (Winter, Spring, Summer, Fall), with weekend/holiday versus workdays accounted for within the questionnaires described in detail in^19^. All response-derived “*time in residence per year”* outcomes were linked to individually reported employment statuses and used to extrapolate the same values for all remaining participants for which employment data was collected.

### Geographic information system-based community type annotation

All consented participants provided an address for the physical building being tested for radon. In some cases, only a Forward Sortation Area (FSA) or full postal code were provided. These locations were converted to the corresponding geo-coordinates (latitude and longitude) using either a Google Maps API service^87^, ArcGIS Pro 3.1.2 Geocode Addresses function (based on ArcGIS World Geocoding Service), or Statistics Canada’s Postal Code^OM^ Conversion File (*PCCF* + *Version 7E, November 2021*) which is based on data licensed from Canada Post Corporation and implemented using SAS 9.4 (a statistical software suite). The resulting geocoordinates were then used to assign a community type based on population size following Statistic’s Canada protocol. The community types (and their population ranges) were cities (≥ 100,000), large towns (30,000–99,999), small towns (1000–2999) and villages, hamlets and isolated areas (1–999). For the purposes of this work, urban community types were defined as cities and large towns, whereas rural community types consisted of small towns and villages, hamlets and isolated areas. Using the most up-to-date (2021 census data) population centre and designated place boundary map files available from Statistic Canada^88^ each location was mapped using the spatial join junction of ArcGIS Pro. A residence was considered to be in a village, hamlet or an isolated area, when it did not intersect a known population centre or designated place, or the designated place or population centre had a population less than 1000 individuals based on the 2021 census data. The other community types were assigned based on their identified location and population size. To determine accurate population sizes, Statistics Canada’s GeoSuite was used^89^. For those places not captured by the 2021 census, the last method was to perform a Google (www.google.com) search of the population centre or designated place name or radon test location to manually determine the population size.

### Groundwater radon analysis

Groundwater samples for radon analysis were collected by, or in direct collaboration with, Alberta Environment and Protected Area’s Groundwater Observation Well Network team between 2019 and 2021. Briefly, groundwater radon samples were collected in 250 mL glass bottles while submerged in a bucket that was overflowing with water to minimize atmospheric contamination. The bottles were filled, several volumes allowed to flush through the bottle, and capped (all underwater). After collection, the samples were maintained at 4 °C until analysis. Radon concentrations in water samples were measured using a Durridge RAD7® with a RADH2O® accessory. Radon analyses were conducted within 24 h of collection and all reported concentrations were corrected for decay over the time that elapsed between collection and analyses. For further information, please see^90^.

### Statistical analysis

Statistical analyses were carried out using MS Excel and GraphPad Prism 9.1.1 (225) (www.graphpad.com), SAS 9.4, SPSS 26, Power BI 2.119.986.0. Data were analysed for log normal or normal distributions. In all cases within this study, residential radon level data were log normally distributed. For an appropriately powered statistical analysis, data were first log transformed then one-way ANOVAs were carried out on the transformed datasets to test differences between groups (e.g., year of construction, occupant age, mSv, etc.). To adhere to best statistical practice, Games-Howell (when groups had n > 50) or Dunnett’s T3 (when groups had n < 50) post-hoc testing was done to correct for multiple comparisons, and used to determine group differences for pairwise comparisons if the ANOVA reached significance.

### Water well information

Well water location information used in this study was obtained from the 2023 ‘Alberta Water Well Information Database’ provided by the Groundwater Information Centre. This can be accessed at^67^. We emphasize that the GIS coordinates for well water locations within this database are accurate to ± 800 m of their exact physical location.

### Supplementary Information

Supplementary Information.

## Data Availability

The de-identified raw data sets generated by the current study are available to researchers following reasonable requests to Dr. Goodarzi, and will require a legally binding data transfer agreement. Data may not be used for private, commercial, or for-profit purposes for any reason.
