# Supplementary material for: Rural communities experience higher radon exposure versus urban areas, potentially due to drilled groundwater well annuli acting as unintended radon gas migration conduits
Source: Sci Rep. 2024 Feb 26;14:3640. doi: 10.1038/s41598-024-53458-6 (PMC10897331; doi:10.1038/s41598-024-53458-6)
Supplement: Supplementary file 1 — Supplementary Information. [file 41598_2024_53458_MOESM1_ESM.pdf]

SUPPLEMENTAL FILE

for

Rural communities experience higher radon exposure versus urban areas, potentially due to drilled groundwater well annuli acting as unintended radon gas migration conduits.

Selim M. Khan[1,2*], Dustin D. Pearson[1,2*], Evangeline L. Eldridge[3], Tiago A. Morais[3], Marvit I. C. Ahanonu[4], M. Cathryn Ryan[3], Joshua M Taron[4], Aaron A. Goodarzi[1,2**]

Author Affiliations:

[1]Robson DNA Science Centre, Department of Biochemistry & Molecular Biology, Charbonneau Cancer Institute, Cumming School of Medicine, University of Calgary, Alberta, Canada; [2]Department of Oncology, Charbonneau Cancer Institute, Cumming School of Medicine, University of Calgary, Alberta, Canada; [3]Department of Earth, Energy and Environment, Faculty of Science, University of Calgary, Alberta, Canada; [5]School of Architecture, Planning, and Landscape, University of Calgary, Alberta, Canada.

*The first two authors contributed equally

**Corresponding author is A. Goodarzi (a.goodarzi@ucalgary.ca)

Property Metric Questionnaire

The questions below are/were asked of all participants following confirmation of informed consent using an online platform compatible with desktop and mobile devices.

The Structure of the survey is generally as follows:

Q0. Question in bold
The limitations on the answer parameters
Answers

Description: (the information displayed for the participant that is pertinent to the question)

Q1. Are you retesting your home after having installed an active/passive soil depressurization system (radon mitigation)

Select one of the following answers

- **Yes**
- **No**
- **Not sure/prefer not to say**

Q2. Relative to the ground outside, what floor of the property will the detector be placed on?
Choose one of the following answers

- Basement/Cellar
- Main/Ground
- First Upper Floor
- Second Upper Floor
- Third Upper Floor
- Fourth Upper Floor
- Fifth Upper Floor
- Sixth Upper Floor
- Seventh Upper Floor
- Eighth Upper Floor
- Ninth Upper Floor
- Tenth (or greater) Upper Floor

Description:

Below are the descriptions to help choose which option fits best if unsure which floor the detector was placed on:

- 0 – Basement/Cellar (any level below outside ground level, often a single storey below main level)
- 1 - Main/Ground (This is the main floor that is essentially level with the ground outside)
- 2 – First Upper Floor (one storey above the ground level outside)
- 3 – Second Upper Floor (two storeys above the ground level outside)

...

(and so on)

Q3. Type of home:

Choose one of the following answers

- Single Detached - Bungalow
- Single Detached - Split Level
- Single Detached - 2 Storey
- Single Detached - 3 Storey
- Semi-Detached - Row House/Townhouse/Side-by-Side
- Semi-Detached - Duplex/Fourplex
- Apartment Block with less than 5 storeys
- Apartment Block with 5 or more storeys
- Trailer/Mobile Home
- Cabin
- Other

Description: Below are descriptions to help you choose the most accurate option for the type of home which the Radon test was completed in:

- 1 - Single Detached - Bungalow (Single Detached indicates that this home is completely separate from other homes, and bungalow means that there is one floor above ground, and one floor below ground)
- 2- Single Detached - Split Level (Single Detached indicates that this home is completely separate from other homes, and a split level means that the floors of the home are staggered. Usually there will be two short sets of stairs, one leading the basement, and one to the upstairs/bedroom level)
- 3- Single Detached - 2 Storey (Single Detached indicates that this home is completely separate from other homes, A two storey home is one that has two full floors above the ground level)
- 4 - Single Detached - 3 Storey (Single Detached indicates that this home is completely separate from other homes, A three storey home is one that has three full floors above the ground level)
- 5 - Semi-Detached - Row House/Side-by-Side/Townhouse (Semi-Detached indicates that the home is partially attached to another home, but there is still a completely separate entrance to the front and back of the home.)
- 6 - Semi-Detached - Duplex/Fourplex (Semi-Detached indicates that the home is partially attached to another home, but there is still a completely separate entrance to the front and back of the home.A duplex is a home that contains two separate apartments or suites. Usually, this will be one suite above ground and one below (the basement suite). A fourplex is one that contains four apartments or suites).
- 7 - Apartment Block with less than 5 storeys (An apartment complex that had less than 5 floors. Usually a smaller complex)
- 8 - Apartment Block with 5 or more storeys (an apartment complex that has 5 or more floors)
- 9 - Trailer/Mobile Home (The home itself likely has wheels and/or can be transported easily)
- 10 - Cabin (This is a smaller home that is likely not located in a bigger town or city, but in a remote location. Cabins are usually found in the woods, mountains, or beaches.)
- 11 - Other (if your home does not match any of these descriptions, then select Other)
- If the home is entirely below ground, please select "Prefer not to say/Not sure". Otherwise please select the option that best applies

Q4. Year of construction?

Only four digit numbers may be entered in this field, could make this a drop down going back to 1800 but projected forward at least until 2025.

Description: The exact year that the construction of this property was completed.

Q5. For the building tested, do you?

Choose one of the following answers

- Own the property
- Rent the property
- Prefer not to say or not sure

Q6. For the building tested, do you live in the property?

Choose one of the following answers

- Yes, full time
- Yes, part time
- No
- Prefer not to say or not sure

Q7. What is the square footage of the ground/main floor, as you would report it on an MLS listing?

Only numbers may be entered in this field.

Q8. What is the square footage of the basement/cellar (if different from above)?

Only numbers may be entered in this field.

Q9. Number of Levels/Storeys above ground?

Only numbers may be entered in this field.

Q10. How high are the ceilings in the basement/cellar?

Choose one of the following answers

- Does not apply
- <8 Feet
- 8 Feet
- 9 Feet
- 10 Feet
- >10 Feet
- Prefer not to say or not sure

Q11. How high are the ceilings on the main/ground floor of this property?

Choose one of the following answers

- <8 feet
- 8 feet
- 9 feet
- 10 feet
- >10 feet
- Prefer not to say or not sure

Q12. How high are the ceilings on the upper floor?

Choose one of the following answers

- Does not apply
- <8 feet
- 8 feet
- 9 feet

- 10 feet
- >10 Feet
- Prefer not to say or not sure

Q13. Basement Type

Choose one of the following answers

- Full Basement
- Full Crawl Space
- Partial Basement
- Partial Crawl Space
- Basement with Crawl Space
- Basement with Multiple Crawl Space
- Full Slab-On-Grade
- Part Basement, Part Slab-On-Grade
- Part Crawl Space, Part Slab-On-Grade
- Prefer not to say or not sure

Q14. Does this property have a walkout basement?

Choose one of the following answers

- Does not apply
- Yes
- No
- Prefer not to say or not sure

Description: A walkout basement is typically observed in a semi-detached or detached family home that, in the lowest storey of the building, has a at least one wall with windows and doors facing the outside where one can typically access the backyard directly from the basement. The remaining three walls face the ground below the main floor of the property.

Q15. What is the Type of Foundation Floor in the lowest level of the property?

Choose one of the following answers

- Poured concrete slab
- Bedrock/Rock
- Earth/Gravel
- Underground Parking
- Prefer not to say or not sure
- Other (fill in box)

Q16. What is the Type of Foundation Walls in the lowest level of the property?

Choose one of the following answers

- Poured concrete
- Cinder Block
- Stone
- Wood
- Prefer not to say or not sure
- Other (fill in box)

Q17. Is there any plumbing in the lowest level of property?

Choose one of the following answers

- No
- Yes, finished plumbing installed (taps, toilets, showers, etc.)
- Yes, but plumbing pipes are only roughed in (not active). Roughed in implies that the pipes themselves are physically present, but they are not actively working or connected to a fixture (such as toilet, sink).
- Prefer not to say or not sure
- Other (fill in box)

Description: Pipes in the basement may be present in the foundation or walls of the basement.

Q18. To the best of your knowledge, the water supply for your home is provided by a:

Choose one of the following answers

- Municipal water supply (i.e. you pay a water utility bill to your City or Town) *this activates the second question*
- Water co-op
- Private Water Well
- Prefer not to say or not sure
- Other (fill in box)

Q19. Please indicate the source of your municipal water supply:

Choose one of the following answers

- Reservoir, canal, river, or lake
- Water wells or groundwater
- Prefer not to say or not sure

Q20. Is there an ACTIVE radon mitigation device with a fan installed in the property?

Choose one of the following answers

- No
- Yes
- Prefer not to say or not sure

Description: An active radon mitigation device includes sub slab depressurization devices that are typically vented outside through the side wall, and are electrically powered with a fan and motor. These are not usually installed at the home construction phase, although in homes built after 2010 may be roughed in. If you have a rough in please do NOT select 'yes' unless an active fan system has been subsequently installed on this (or in any other location).

Q21. Is there a PASSIVE radon mitigation stack installed in the property?

Choose one of the following answers

- No
- Yes
- Prefer not to say or not sure

Description: Passive stack radon suppression measures are being installed in some newer homes in Canada at the build phase, and potentially can be retrofitted to older properties. These are generally vented up through the roof and do NOT have an electrically powered fan.

Q22. Does this property have a Sump Pump?

Choose one of the following answers

- No
- Yes, and it is airtight
- Yes, and it is not airtight
- Yes, but not sure about air tightness
- Prefer not to say or not sure

Description: Older sump pumps (pre 2010 build code) in Canada may not be airtight, and thus vent soil gasses into the property. Newer sump pumps are required to be airtight.

Q23. List of foundation (floor or wall) disruptions

Select all that apply

- There is a ground water well (either in use, or decommissioned) located inside your property.
- Can you see any gaps or holes in the concrete foundation under the furnace unit (perhaps with visible gravel or loose rock showing)?
- Is there any dried concrete residue at the base of your original furnace unit AND/OR return air duct?

Description:

- Water wells: Older/rural/remote properties may have a water well located within the building perimeter, which can affect the properties' air dynamics. The well may be in use (as a source of water) or not and decommissioned.
- Gaps or Holes under furnace: In some parts of Canada, the furnace may be hung from the ceiling at the time of construction before the concrete floor is poured. Often, the concrete underneath the furnace unit does not entirely "close up" leaving a visible gap. This is often visible using a flashlight if you look under your furnace, or temporarily removing the lower housing.
- Dried Concrete on furnace: In some parts of Canada, the furnace may be hung from the ceiling at the time of construction before the concrete floor is poured. If this is the case, there may be specks or lumps of dried concrete left on the furnace if it is the original unit, and/or on the return air duct (even if the furnace is newer). You may be able to see these or feel them if you run your hand lightly over the units.
